# Predicting Pulmonary Function From the Analysis of Voice: A Machine Learning Approach

**DOI:** 10.3389/fdgth.2022.750226

**Published:** 2022-02-08

**Authors:** Md. Zahangir Alam, Albino Simonetti, Raffaele Brillantino, Nick Tayler, Chris Grainge, Pandula Siribaddana, S. A. Reza Nouraei, James Batchelor, M. Sohel Rahman, Eliane V. Mancuzo, John W. Holloway, Judith A. Holloway, Faisal I. Rezwan

**Affiliations:** ^1^Human Development and Health, Faculty of Medicine, University of Southampton, Southampton, United Kingdom; ^2^Department of Computer Science and Engineering, Bangladesh University of Engineering and Technology, Dhaka, Bangladesh; ^3^Department of Information and Electrical Engineering and Applied Mathematics/DIEM, University of Salerno, Fisciano, Italy; ^4^Peter Doherty Institute, The University of Melbourne, Melbourne, VIC, Australia; ^5^Hunter Medical Research Institute, The University of Newcastle, Newcastle, NSW, Australia; ^6^Department of Respiratory Medicine, John Hunter Hospital, Newcastle, NSW, Australia; ^7^Postgraduate Institute of Medicine, University of Colombo, Colombo, Sri Lanka; ^8^Clinical Informatics Research Unit, University of Southampton, Southampton, United Kingdom; ^9^Robert White Centre for Airway Voice and Swallowing, Poole Hospital, Poole, United Kingdom; ^10^Medical School, Universidade Federal de Minas Gerais, Belo Horizonte, Brazil; ^11^National Institute for Health Research Southampton Biomedical Research Centre, University Hospital Southampton, Southampton, United Kingdom; ^12^Clinical and Experimental Sciences, Faculty of Medicine, University of Southampton, Southampton, United Kingdom; ^13^MSc Allergy, Faculty of Medicine, University of Southampton, Southampton, United Kingdom; ^14^Department of Computer Science, Aberystwyth University, Aberystwyth, United Kingdom

**Keywords:** pulmonary function, FEV_1_, speech, breathe, machine learning, human voice, asthma

## Abstract

**Introduction:**

To self-monitor asthma symptoms, existing methods (e.g. peak flow metre, smart spirometer) require special equipment and are not always used by the patients. Voice recording has the potential to generate surrogate measures of lung function and this study aims to apply machine learning approaches to predict lung function and severity of abnormal lung function from recorded voice for asthma patients.

**Methods:**

A threshold-based mechanism was designed to separate speech and breathing from 323 recordings. Features extracted from these were combined with biological factors to predict lung function. Three predictive models were developed using Random Forest (RF), Support Vector Machine (SVM), and linear regression algorithms: (a) regression models to predict lung function, (b) multi-class classification models to predict severity of lung function abnormality, and (c) binary classification models to predict lung function abnormality. Training and test samples were separated (70%:30%, using balanced portioning), features were normalised, 10-fold cross-validation was used and model performances were evaluated on the test samples.

**Results:**

The RF-based regression model performed better with the lowest root mean square error of 10·86. To predict severity of lung function impairment, the SVM-based model performed best in multi-class classification (accuracy = 73.20%), whereas the RF-based model performed best in binary classification models for predicting abnormal lung function (accuracy = 85%).

**Conclusion:**

Our machine learning approaches can predict lung function, from recorded voice files, better than published approaches. This technique could be used to develop future telehealth solutions including smartphone-based applications which have potential to aid decision making and self-monitoring in asthma.

## Introduction

Asthma is a common respiratory condition that affects 235 million people worldwide (1). Around 5.4 million people in the UK are currently receiving treatment for asthma, ~1 in 11 children and 1 in 12 adults (2). Every 10 s, at least one person is facing a potentially life-threatening asthma attack in the UK, and on an average, three people die from it daily, regardless the effective treatments developed in recent years (3). Appropriate, effective management and treatment for asthma is therefore of vital importance.

Many different techniques can monitor the complex nature of asthma, including subjective symptom assessments, lung function testing, and measurement of biomarkers. Regular monitoring of asthma can help patients receive appropriate treatment in time, which can help to reduce symptoms, frequency of exacerbation, and risks of hospitalisation. The ability to monitor asthma and modify treatment appropriately could help to reduce both disease morbidity and the economic cost of treatment. Identifying symptoms via questionnaire and lung function measurement via spirometry identifying of biomarkers (e.g. exhaled nitric oxide or sputum eosinophils) can all be used in regular monitoring of asthma (4). In practise, however, the combination of these is impractical in community-based care due to expense and/or complexity.

Self-monitoring of asthma has the potential to play an important role in empowering the patient and maintaining disease control; such monitoring needs to be simple, convenient, and accurate. Equipment such as smart spirometers and, accompanying smartphone apps used to record peak expiratory flow rates (PEFR) and provide reminders to manage asthma more efficiently are currently available to simplify self-monitoring (5). However, smart spirometers are still expensive for personal use. As more people use smartphones, an application measuring lung function that could alert patients to modify their treatment without the need for a spirometer would be a convenient and inexpensive way to monitor asthma, particularly in Lower- and Middle-Income Country (LIMC) settings.

At present, assessment of the ability to speak and the sounds associated with breathing are a recognised part of an assessment of asthma severity, such as: “speaking full sentences” to “unable to speak at all” together with wheeze on auscultation (6–8). Although no standardised assessment or quantitative measures of these features have been developed, the effects on speech and breathing patterns and sounds due to increased airway resistance are noticeable in acute asthma (9). Thus, pitch from speech and quality of the breathing sound can potentially be utilised as surrogate measures of symptoms and/or to predict lung function, which can then be used to monitor asthma.

Three kinds of sounds have been analysed to predict lung function using machine learning techniques: (1) lung and breathing sounds from the chest, (2) symptom-based sounds (such as a cough sound), and (3) voice sounds. Quantitative breath sound measurements, such as Vibration Response Imaging (VRI), have been used to predict postoperative lung function (10, 11). Cough and wheeze sound-based analyses have been shown to have potential in predicting spirometer readings (12–14).

In parallel to symptom-based sounds (such as a cough sound), there are a number of studies, which involve voice sounds only. A recent review identified 20 studies to date. It confirmed the idea of respiratory function correlating significantly to phonation sound. Some of these studies showed that voice evaluation might allow recognition of asthma contributing to voice dysfunction subjected to lung function (15). However, most of these studies required the use of specialised instruments and software to quantify specialised phonetic sounds. Using machine learning techniques, one of these studies showed that sustained phonation of the vowel sound demonstrated potential utility in the diagnosis and classification of severity of asthma (16).

Assessing the quality of sound produced by an asthma patient, primarily via speech, is a common way to assess acute asthma. We have previously demonstrated that recorded speech correlates well with lung function during induced bronchoconstriction (17). To date, only two studies have utilised machine learning techniques to predict lung function from the recorded voice. Saleheen et al. proposed a convenient mobile-based approach that utilises a monosyllabic voice segment called “A-vowel” sound or “Aaaa…” sound from voice to estimate lung function (18). Chun et al. proposed two algorithms for passive assessment of pulmonary conditions: one for detection of obstructive pulmonary disease and the other for estimation of the pulmonary function in terms of ratio of forced expiratory volume in 1 s (FEV_1_) and forced vital capacity (FVC) also denoted as FEV_1_/FVC and percentage predicted FEV_1_ (FEV_1_%) (19). However, these studies showed moderate performance and did include comparison with previous studies.

This study proposes a new methodology to predict lung function from recorded speech using machine learning techniques to monitor asthma. Bronchoprovocation tests were given to participants to help diagnose asthma, and their voices were recorded for 1 min while the subjects read standard texts with lung function measured. This study aims to identify features from recorded speech files that correlate with measured lung function. We subsequently use those features to predict lung function, potentially enabling identification of deterioration of asthma control via a smartphone application in the future.

## Materials and Methods

### Dataset

Twenty-six non-smoking, clinically stable subjects, with physician-diagnosed mild atopic asthma, were recruited on step one of treatment according to 2012 GINA guidelines (20). The study was approved by the local ethics committee (number 12/EE/0545), by the Medicines and Healthcare Products Regulation Agency (MHRA) (MHRA number 11709/0246/001-0001) (21). The study was performed in compliance with the protocol and additional methodologic details provided in the Supplementary Material. All participants underwent a standardised inhaled methacholine challenge (21) and after each challenge dose, the participant read a standardised text for 30 s into a digital recorder fitted with an external microphone set at 10 cm from the mouth (Olympus DM450 Speech Recorder with Olympus ME34 Microphone, Tokyo, Japan). After each dose of the bronchial challenge, the voice of each subject was recorded, and lung function was measured as FEV_1_% predicted. Spirometry was performed with a dry bellows spirometer (Vitalograph, UK) and the best of at least three successive readings within 100 ml of each other was recorded as the FEV_1_ in accordance with established guidelines (22). In total 323 voice recorded sound files with their associated FEV_1_% were recorded for these 26 subjects. Details of the method is shown in the Supplementary Methods section. Additionally, an overview of the basic biological attributes (i.e., sex, height and weight) of these samples is reported in Supplementary Table 1.

### Separation of Breathing and Speech Segments From Sound Files

An exploratory analysis was carried out on a segment of speech and breathing separately in the frequency domain and considerable differences were noticed in the spectrograms generated by librosa (23) (Supplementary Figure 1). The parts of the sound file containing breathing and speech were separated from five randomly selected sound files using Audacity software (24). Features (including roll-off at 85/95%, spectral fitness, root mean square energy, zero crossing rate, spectral centroid, spectral bandwidth, spectral contrast, spectral flatness, mean amplitude, and mean breath cycle duration) (described in Supplementary Methods) were extracted for individual breathing and speech segments, using the librosa tool. These features were analysed to explore differences between breathing and speech segments and determine the appropriate thresholds to separate breathing and speech segments.

### Feature Extraction

After analysing the values for individual speech and breathe segments, only five features (Spectral contrast, Roll-off at 95%, Root mean squared energy, Spectral bandwidth, and Mean amplitude) showed substantial differences between breathing and speech segments (Supplementary Figure 2). Based on the observed information, using a threshold, these 5 features were defined (Supplementary Table 2), which separate the breathing and speech segments from all available sound files.

All extracted features were Min-max normalised. As there was a low number of features, it was impossible to utilise a feature engineering method to identify informative features. The use of Pearson correlation coefficient calculated the correlation between the features and FEV_1_%.

### Predictive Model Development

Training and testing samples were separated randomly at a ratio of 70%:30%, respectively (i.e., the training dataset contained 70% of the samples, whereas the testing dataset kept the remaining 30% of the samples). This defined the following three types of predictive models:

Model_1_: A regression model to predict FEV_1_% predicted based on the features extracted from recorded sound data. The techniques and the feature set for which this model performs best were applied for the other following models. The performances of these models are reported in terms of Root Mean Square Error (RMSE) and mean absolute error (MAE).

Model_2_: A multi-class classification model to predict the severity of abnormality of lung function according to American Thoracic Society (ATS) grades (as defined in Supplementary Table 3) (25).

Model_3_: A binary classification model to predict FEV_1_% classified either as normal or abnormal based on the ATS definition of abnormal lung function (Supplementary Table 3), where lung function is normal if FEV_1_% > 80%, otherwise lung function is abnormal.

Three machine learning algorithms following other studies (19, 26, 27) available in this contextual domain and/or in other similar domains, i.e., Random Forest (RF), Support Vector Machine (SVM) (using Radial Basis Function kernel), and Linear Regression/Logistic Regression (for the binary task), were implemented to develop the predictive models. Training the models was undertaken on the “training set,” and 10-fold cross-validation was used to measure the models training performances. The models use default values of hyperparameters, and tuning did not show any improvements over default parameters. Finally, the models were run on the testing samples to assess the final performances.

Further basic biological factors, including sex, height and weight of the subjects were added as features in addition to the features extracted from the sound file as mentioned above to develop three additional models (denoted as Model_1P_, Model_2P_, and Model_3P_, respectively for Model_1_, Model_2_, and Model_3_).

Initially, we investigated the effect of features extracted from speech and breathing parts both individually and combined on lung function using Model_1_. We also explored the issue of imbalances of distribution of lung function in random partitioning and performed balanced partitioning of training and test samples as follows:

1. Using intervals of 5% on the FEV_1_% values 15 groups were prepared. For example, groups are 51–55%, 56–60%, 61–65% etc.
2. Based on the groups sample distribution was prepared.
3. Based on the sample distribution in each group balanced training and test sets were prepared, such that training and test sets followed the same distribution.

## Results

Supplementary Table 4 shows the severity of abnormal lung function among 323 data samples. It is evident that 72.14% of samples exhibited normal lung function during corresponding recording and the rest (27.86%) exhibited abnormal lung function.

### Feature Extraction From Recorded Voice Files

Fourteen breathing segments and nine speech segments were retrospectively extracted from the sound files (Supplementary Table 5). The results show no correlation between the features and FEV_1_% (Supplementary Figure 3).

### Lung Function Prediction in Terms of FEV1% (Model_1_)

#### Effect of Speech and Breathing Features on Prediction

**Figure 1:**
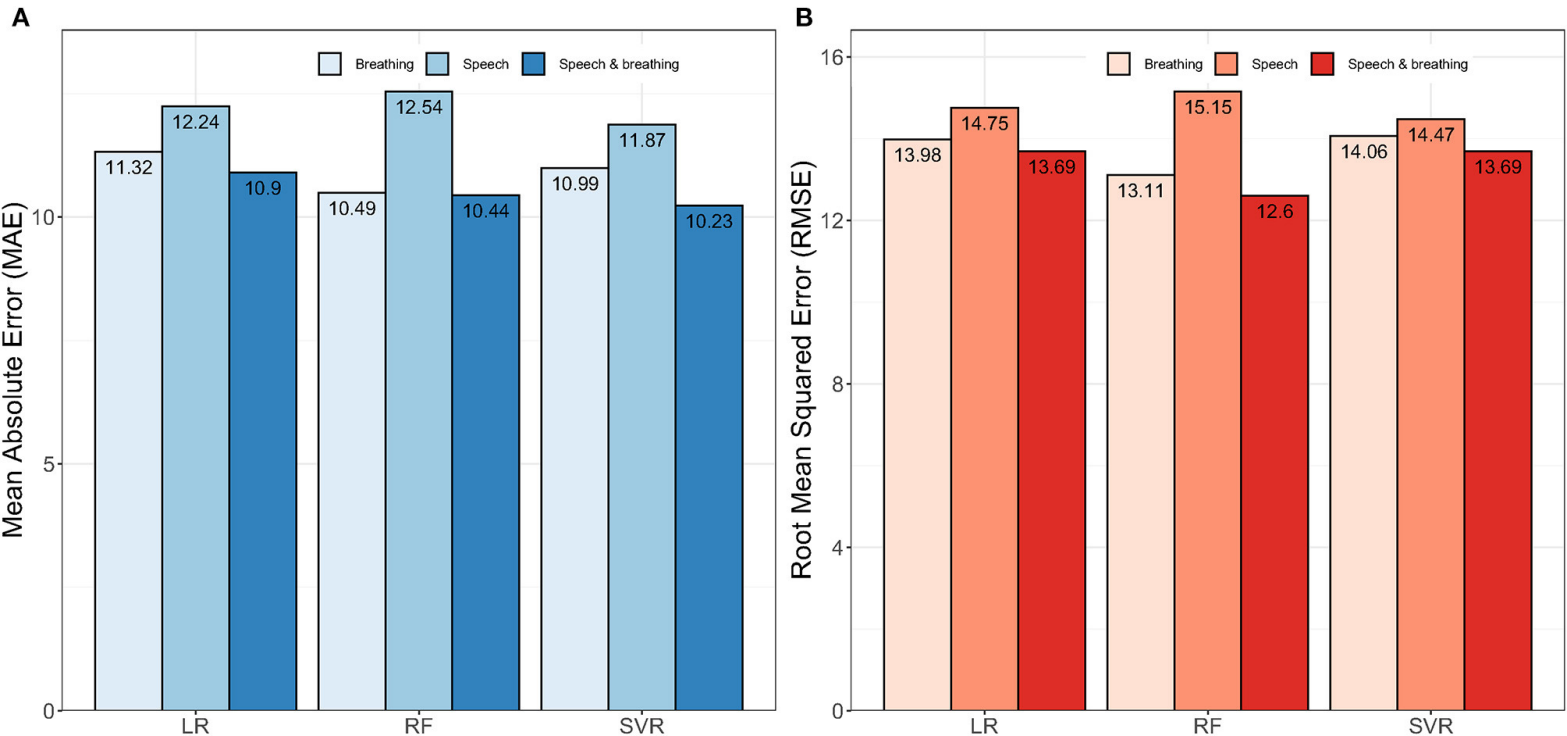
Impact of speech and breathing features individually and combined on model development. **(A)** Shows the performances of the models in terms of mean absolute error (MAE) and **(B)** presents the performances of the models in terms of root mean squared error (RMSE). Here, LR, Linear Regression; RF, Random Forest; SVR, Support Vector Regression.

Initially, we explored the ability to predict lung function from extracted features of speech and breath both individually and in combination. Regression models developed using the combined features from speech and breathing to predict FEV_1_%, showed lower mean absolute error (MAE) than that of models developed from features from speech and breathing separately (Figure 1A). The RF model [Model_1(RF)_] performed better in comparison to all other algorithms (the lowest Root Mean Square Error, RMSE = 12·59) (Figure 1B).

#### Effect of Balanced Partitioning of the Training and the Testing Sets

The samples were not uniformly distributed amongst the ranges of FEV_1_% (Supplementary Figure 4). The frequency of the samples is the highest around 100 of the FEV_1_% values and no sample was found with the FEV_1_% ≤ 50. As a result, samples were not uniformly distributed among the ranges of FEV_1_% values. Therefore, when the training and test samples were divided randomly, the pattern in the training dataset may not follow the pattern in the test dataset (Supplementary Figure 5A). The balanced separation of training and test samples shows a similar pattern of the samples among each range of FEV_1_% (Supplementary Figure 5B).

Balanced partitioning of the training and the test sets led to improved performance compared to random partitioning. This is evident for all regression models, where balanced partitioning shows lower RMSE and MAE scores in comparison to the random partition model (Supplementary Figure 6). Again, the RF based model [Model_1(RF)_,] performed better than other models (RMSE = 12·51 and MAE = 9·83).

#### Effects of the Phenotypes on the Predictive Models

**Figure 2:**
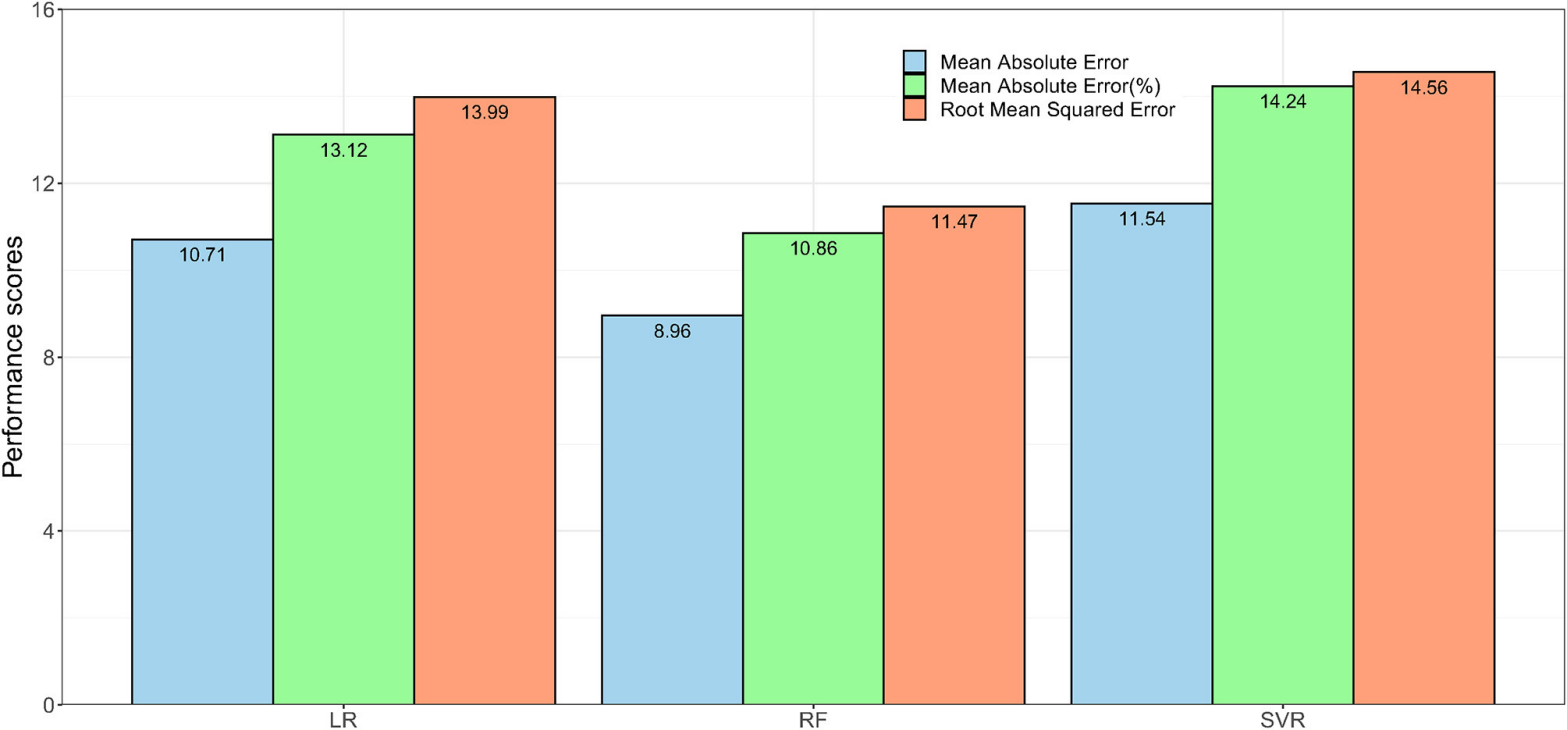
The performance of the regression models. Model_1P_ used extracted features from speech and breath parts with sex, height and weight to predict lung function in terms of FEV_1_%. Here, LR = Linear Regression, RF = Random Forest, and SVR = Support Vector Regression.

The performance of the models when biological factors were added are shown in Figure 2. The RF based algorithm performed better with MAE (%) score of 10.86 and RMSE score of 11·47 in comparison to other algorithms. Supplementary Table 6 shows the comparison of Model_1(RF)_, and Model_1P(RF)_ for predicting FEV_1_%. Model_1P(RF)_ showed better predictive performance than that of Model_1(RF)_.

### Severity of Abnormality of Lung Function Prediction (Model_2_)

The performance of Model_2_ and Model_2P_ in predicting lung function severity from the sound files, is shown in Table 1. Model_2(SVC)_ predicted abnormal lung function with 71% accuracy, while Model_2P(SVC)_ predicted this with 73.2% accuracy.

**Table 1:** Comparison of the performances of Model_2_ and Model_2P_ in predicting the severity of abnormality of lung function.

**Algorithms**	**Model_**2**_**	**Model_**2P**_**
Linear Regression	0.64	0.66
Random Forest	0.68	0.71
Support Vector Classifier	0.71	0.73

*Model_2_ used only features extracted from breath and speech parts and Model_2P_ included biological factors (sex, weight, and height) with the features in multi-class classification models. Predicted multi-classification was defined based on severity of abnormality of lung function graded by American Thoracic Society (ATS). Here, performance is represented in terms of accuracy*.

### Normal *vs*. Abnormal Lung Function Prediction (Model_3_)

**Figure 3:**
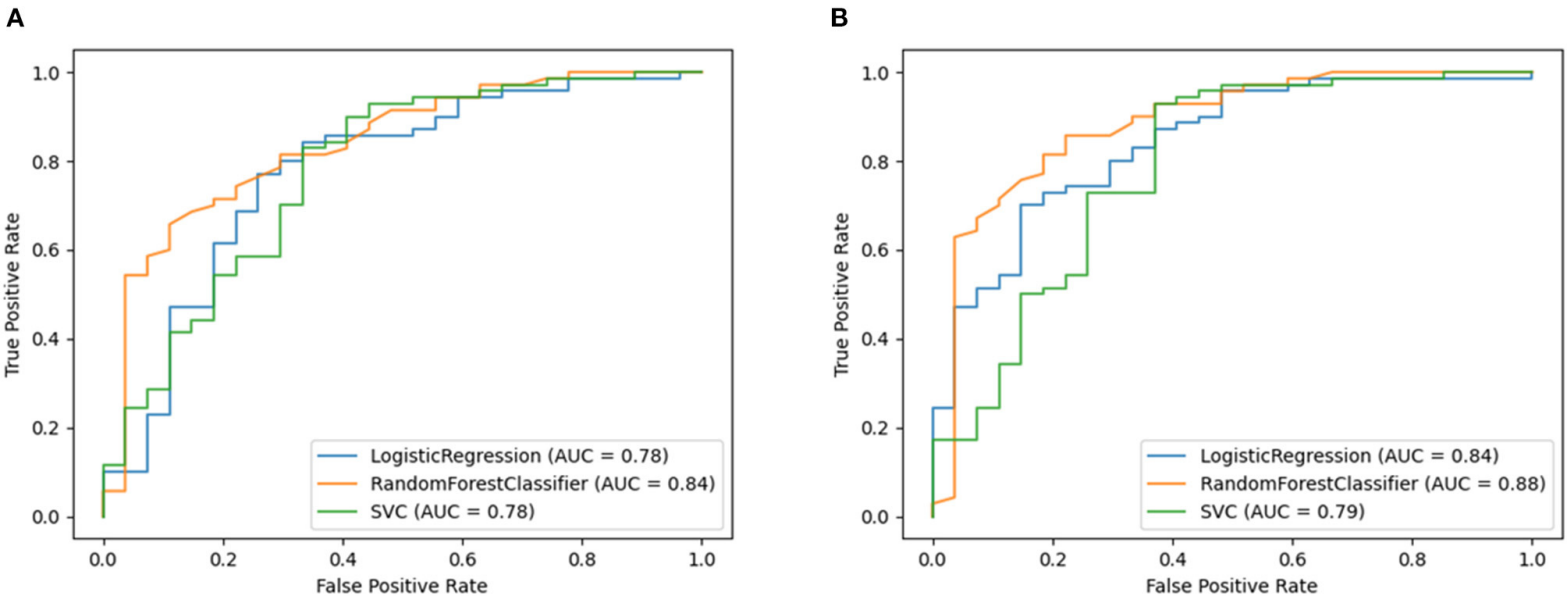
Receiver operating characteristic curve plots of Model_3_ and Model_3P_. Model_3_ used only features extracted from breath and speech parts and Model_3P_ included biological factors (sex, weight, and height) with the features in binary class classification models. Predicted binary classification was defined based on FEV_1_% classified either as normal (FEV_1_% < 80%) or abnormal (FEV_1_% ≥ 80%) based on the ATS definition of abnormal lung function. These plots show the area under Receiver Operating Characteristic curve of model's showing performance for predicting normal vs. abnormal lung function. **(A)** showing the ROC curve for Model_3_ and **(B)** showing the ROC curve for Model_3p_.

The performance of the models (without and with biological factors) in predicting normal vs. abnormal lung function are shown in detailed in Supplementary Tables 7, 8. The best performance (without adding biological factors) was observed for the RF model, Model_3(RF)_, with 80% accuracy and 79% F_1_-score. The RF based model clearly performs better (AUC = 0.84) than the other models (Supplementary Tables 7, 8; Figure 3A).

This held true when adding physical attributes, with the RF-based model again showing the best performance (accuracy = 85%, F1-score = 84%, and AUC = 88% AUC (Supplementary Tables 9, 10, Figure 3B).

## Discussion

This study focused on predicting lung function from recorded voice sounds in three ways and has developed a predictive model (for FEV_1_%), which can be utilised in real-time applications for asthma management. A model to predict the severity of abnormal lung function as defined by the ATS (22) was also developed, as well as a model to predict normal vs. abnormal lung function (i.e., FEV_1_% ≤ 80). By detecting abnormal lung function, this can be used to prompt the patient to take appropriate action to manage their condition.

A threshold-based mechanism was defined to separate the breathing and speech features from the recorded sound files and 23 features extracted to develop the predictive models. Using both breathing and speech features in combination improved the performance of the predictive models. This is consistent with standard clinical practise to identify acute asthma by listening to both speech and breathing patterns.

Handling partitioning of the training and the testing dataset is an important factor in developing the prediction model. Considering the American Thoracic Society Grades for the severity of a Pulmonary Function Test Abnormality (28), this study utilised a balanced partitioning technique for predicting FEV_1_% for asthma patients and, consequently, the model's performance was improved in comparison to random partitioning given the imbalance in the available data set.

Initially, the RF based predictive models showed better performance in comparison to other models except for the prediction of severity of abnormality of lung function. The RF based models predicted FEV_1_% with lower RMSE and MAE, and abnormality in lung function with high accuracy. In contrast, SVM predicted severity of lung function with higher accuracy compared with that of RF and LR based models. Given the feature space in this study is not highly dimensional (with only 26 features in total), these results are consistent with previous studies that have reported better performance of RF based models when working with a limited number of features (14, 19). Generally, SVM is applied to highly dimensional space for best results. In addition, the correlational matrix showed no strong correlation of any feature with FEV_1_%, and RF performs better with non-linear problems.

Furthermore, due to their nature, RF models are less likely to overfit. While most of the scores of the RF-based models are quite reasonable, the sensitivity of the RF based classifier to predict abnormality of lung function was not high (sensitivity = 44%). This could be due to the imbalanced distribution of the available samples to normal vs. abnormal lung function (~3:1). Although RF based models (Model1 and Model_3_) showed better performance, the SVM based model performed well on predicting severity of lung function (Model_2_). This is possibly due to grouping samples (i.e., grouping of FEV_1_%) based on the severity of abnormality of the lung function and heterogeneous distribution of the samples into these groups (e.g., the samples with normal and with moderate to severely abnormal lung functions are 72.14% and 2.79% respectively in the dataset).

Adding biological factors (sex, height and weight), to the model, along with the features extracted from speech and breathing, improved the performances of the models. This improvement was observed for all three methods (RF, SVM and LR) used in this study.

Breathing becomes more difficult for people with obstructive pulmonary disease due to increased airway resistance. As their pulmonary symptoms worsen, they frequently notice increased breathlessness and may have higher respiratory rates (29). Previous predictive models for respiratory disease severity have used many pulmonary features, such as Mean Breath Cycle Duration and Breath Number, that relate to airway resistance in patients with pulmonary disease (e.g. asthma). For example, an earlier study reported a higher rate of increase in the intensity of the sound for equal increments in flow rate in chronic bronchitis and asthma than in healthy subjects (30). The pulmonary features extracted in this study that predict lung function are in line with these previous observations. However, the inclusion of additional features in the prediction models such as Roll off 95%, Mean Amplitude, Spectral Bandwidth etc., are also important in the prediction performance of our models.

Only two recent studies have used voice sounds to predict lung function. Saleheen et al. extracted the “A-vowel” segments from the voice sound and then extracted features from the ‘A-vowel' sounds and predicted lung function in terms of the FEV_1_/FVC ratio (18). Due to the unavailability of FVC values in this study, it is not possible to directly compare results. Chun et al. developed models to predict lung function in terms of the FEV_1_/FVC ratio and FEV_1_% (19). Their reported prediction efficiency in terms of MAE (%) score is 20.6%, which is significantly large for any regression problem. The RF based regression model reported in this study achieved a MAE (%) score of 10.86%, a significant improvement over that of Chun et al.

This pilot study has limitations, including the limited sample size of matched audio and lung function measurements and the range of machine learning algorithms utilised to develop the predictive models. To overcome this, the balanced partitioning technique was applied. The performance of the predictive regression model in estimating FEV_1_% values was reasonable, and better than previous studies found in the literature, in addition to being able to predict normal vs. abnormal lung function and the severity of abnormality of lung function. To avoid overfitting and increase the likelihood of the model being generalizable, 10-fold cross validation was used during training of the model. Furthermore, no feature selection method was applied to identify important features among the 23 features. However, due to the limited number of features, this study did not consider feature engineering. Future work utilising a large set of samples, together with an independent validation sample will allow the predictive model to be better generalised and allow validation. A recent study included Mel Frequency Cepstral Coefficient (MFCC) value as a feature to predict COVID-19 subjects from a forced-cough cell-phone recording (31). In contrast, our study has used 23 features excluding MFCC due to the nature (breathing and speech) of pattern-finding for acute asthma prediction from sound files. MFCC represents a full signal at a time in the signal processing. On the other hand, 23 features extracted here present the micro-information of different parts of a signal (e.g., breathing chunks and speech chunks of a voice sound file). However, the application of MFCC along with these features may have the potential to further improve the predictions.

This study used the sound recordings available from a previous study (17). While comparing the performances between recorded speech between external microphone and smartphone will be helpful to understand the future use of this method using smartphone, this was beyond the scope of this study. As we wanted to establish the proof of concept that lung function can be predicted from voice recordings, a future work is warranted to predict lung function using recorded voice from the smartphone.

Asthma puts pressure on health services due to the associated cost and workforce required to treat and care for the people with the condition. Therefore, regular monitoring and early intervention can help control the disease, reducing hospital admissions and economic and social burden to the patient and healthcare system. The predictive models developed in this study can be implemented in smartphone applications offering a convenient and straightforward way to predict lung function. Embedding the algorithm in an app for self-monitoring asthma will potentially enable patients to achieve improved symptom control, via early treatment of exacerbations. The demonstration that it is possible to use machine learning as a surrogate measure for underlying lung function has the potential to lead to the development of telemedicine solutions to improve early diagnosis, reduce unplanned hospital admissions and mortality for respiratory disease through supporting clinical decision making and patient self-monitoring. Further development of this AI speech/breathing technology will allow assessment of lung function in a cross-cultural, language-independent manner in order to assist in the remote monitoring of patients with a range of chronic lung conditions, including asthma, COPD, and pulmonary fibrosis.

## Data Availability Statement

The raw data supporting the conclusions of this article will be made available by the authors, without undue reservation.

## Ethics Statement

The study was 120 approved by the Local Ethics Committee (number 12/EE/0545), by the Medicines and Healthcare 121 Products Regulation Agency (MHRA) (MHRA number 11709/0246/001-0001).

## Author Contributions

JAH and FR conceptualised the study. JWH, JAH, and FR designed the study. NT and CG collected the data. MA, AS, and RB analysed data. MA, JAH, and FR contributed to data interpretation and drafted the article. NT, CG, PS, SN, JB, MR, EM, and JWH critically reviewed the article. All authors reviewed the literature, read, and approved the final article.

## Funding

The study was funded by the Asthma, Allergy and Inflammation Research Charity, Southampton, UK. MA is a Commonwealth Scholar, funded by the UK government.

## Conflict of Interest

The authors declare that the research was conducted in the absence of any commercial or financial relationships that could be construed as a potential conflict of interest.

## Publisher's Note

All claims expressed in this article are solely those of the authors and do not necessarily represent those of their affiliated organizations, or those of the publisher, the editors and the reviewers. Any product that may be evaluated in this article, or claim that may be made by its manufacturer, is not guaranteed or endorsed by the publisher.
