# Supplementary material for: Predicting Pulmonary Function From the Analysis of Voice: A Machine Learning Approach
Source: Front Digit Health. 2022 Feb 8;4:750226. doi: 10.3389/fdgth.2022.750226 (PMC8861188; doi:10.3389/fdgth.2022.750226)
Supplement: Supplementary file 1 [file Data_Sheet_1.pdf]

Supplementary Materials: Predicting pulmonary function from the analysis of voice: a machine learning approach

1 Supplementary Method

1.1 Allergen challenge:

An allergen challenge test involves directly exposing a person to a substance to try to find out whether the person is allergic to it.

1.2 Bronchial challenge

A bronchial challenge test is a medical test used to assist in the diagnosis of asthma. The person's normal lung function will be measured first (also known as his/her baseline breathing). Then he/she will start the test. During the test, he/she will be asked to inhale doses of methacholine, a drug that can cause narrowing of the airways. If his/her lung function drops by 20% or more from his/her baseline, he/she may have asthma. The test takes about 90 minutes. The breathing test will be repeated after each dose of methacholine to measure the degree of narrowing or constriction of the airways, therefore, multiple test results will be produced from a person.

1.3 Forced Expiratory Volume (FEV)

Forced expiratory volume (FEV) measures how much air a person can exhale during a forced breath. The amount of air exhaled may be measured during the first second (FEV_1), second seconds (FEV_2), and/or third seconds (FEV_3) of the forced breath. A lower than normal FEV1 reading suggests that the person may be experiencing a breathing obstruction. In other words, a person who has asthma or COPD has a lower FEV_1 result than a healthy person. The normal values for FEV1 vary from person to person. They are based on standards for an average healthy person of their age, race, height, and gender. Each person has their own predicted FEV1 value based on these factors mentioned above. We can get our predicted normal value with a spirometry calculator. If we know our FEV_1 value already, we can enter it as well, and the calculator will tell us what percent of the predicted normal value our result is.

FEV_1 can be converted to a percentage of the predicted normal value, termed as $FEV_1\%$. It is defined as follows:

$$\mathrm{FEV_1\%} = \left(\frac{\mathrm{FEV_1}}{\mathrm{Predicted\ FEV_1}}\right) * 100$$

1.4 Reading material for participants

Instructions: please read the following text at the same volume as if you were talking on the telephone. If you reach the end of the text then please start again from the beginning.

When we got to the strange house it began to snow in quite a different way. A mass of tired old clouds opened and flung snow at us, all of a sudden and just anyhow. They weren’t ordinary snowflakes – they fell straight down in large sticky lumps, they clung to each other and sank quickly and they weren’t white, but grey. The whole world was as heavy as lead. Mummy carried in the suitcases and stamped her feet on the doormat and talked the whole time because she thought the whole thing was such fun and that everything was different. But I said nothing because I didn’t like this strange house. I stood in the window and watched the snow falling,

and it was all wrong. It wasn't the same as in town. There it blows black and white over the roof or falls gently as if from heaven, and forms beautiful arch over the sitting-room window. The landscape looked dangerous too. It was bare and open and swallowed up the snow, and the trees stood in black rows that ended in nothing. At the edge of the world there was a narrow fringe of forest. Everything was wrong. It should be winter in town and summer in the country. Everything was topsy-turvy. The house was big and empty, and there were too many rooms. Everything was very clean and you could never hear your own steps as you walked because the carpets were so big and they were as soft as fur.

(Source: Tove Jansson (2006). *A winter book.* London: Sort Of Books.)

2 Details of the features

1. **Roll-off at 85%/95%:** It represents the value in frequency below which is possible to find the 85%/95% of the total frequencies.
2. **Spectral flatness:** It is a measure to quantify how much noise-like a sound is, as opposed to being tone-like.
3. **Root mean squared energy:** The energy of a signal corresponds to the total magnitude of the signal. For audio signals, that roughly corresponds to how loud the signal is. The energy in a signal is defined as:

$$\boldsymbol{Energy} = \sum_{1}^{N} |\boldsymbol{x}(\boldsymbol{n})|^2$$

   where x[1] is a frame of a signal and n is the length of a frame.

   The root mean squared energy in a signal is defined as:

$$\sqrt{\frac{1}{N}\sum_{1}^{N} |\boldsymbol{x}(\boldsymbol{n})|^2}$$

4. **Zero Crossing Rate:** It indicates the number of times that a signal crosses the horizontal axis.
5. **Spectral centroid:** It is a measure used in digital signal processing to characterize a spectrum. It indicates where the centre of mass of the spectrum is located. Perceptually, it has a robust connection with the impression of brightness of a sound.
6. **Spectral bandwidth:** It is a measure of the extent of the spectrum. It is the wavelength interval in which a radiated spectral quantity is not less than half of its maximum value.
7. **Spectral contrast:** It considers the strength of spectral peaks and spectral valleys in each sub-band separately.
8. **Spectral Flatness:** It is defined as the ratio of the geometric mean to the arithmetic mean of a power spectrum.
9. **Mean amplitude:** It is the mean amplitude of various frequencies present in a sound signal. It is measured using Short Term Fourier transformation (STFT) of a signal. STFT converts a signal in such a way so that we can understand the amplitude of given frequency at a given time.
10. **Mean breath cycle duration:** Breath cycle duration is the duration from one breath chunk to the next breath chunk. The mean breath cycle duration is the mean of all breath cycle durations.

3 Supplementary Figures

(a) Power spectogram of a breath segment

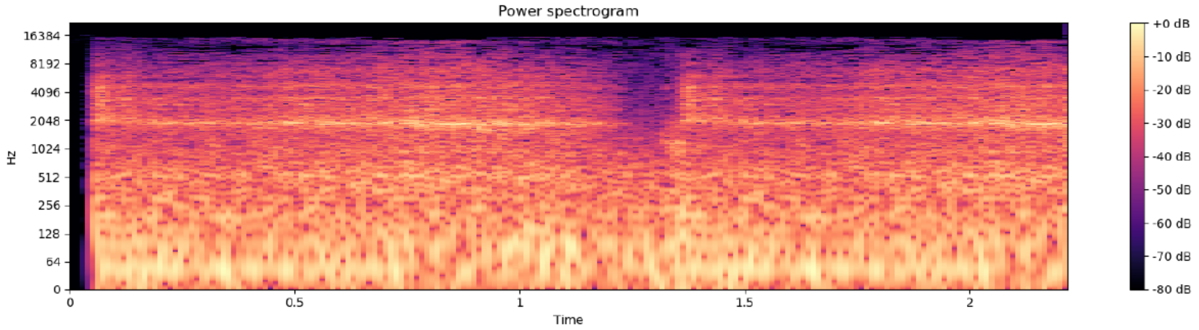

(b) Power spectogram of a speech segment

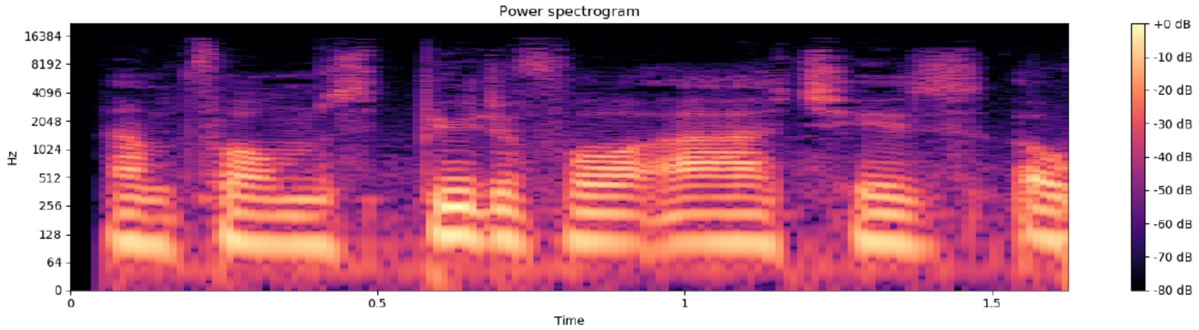

Figure S1: Power spectrograms of a breathing chunk, and of a speech chunk from a sound file. It presents two power spectrograms: [a] Power spectrogram of a breathing chunk, [b] Power spectrogram of a chunk of a speech. In these spectrograms, we can observe that the breath segment exhibits higher frequencies, and the power of the signal is distributed more evenly throughout the time interval.

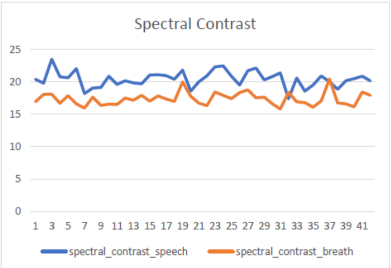

(a) *Spectral contrast graph for breaths and speeches chunks*

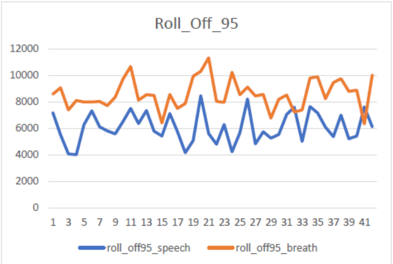

(b) *Roll off 95% for breaths and speeches chunks*

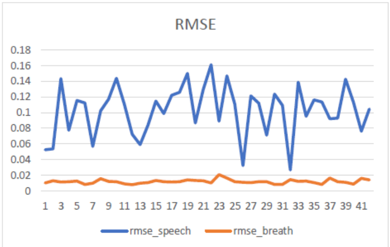

(c) *Root-mean-square deviation for breaths and speeches chunks*

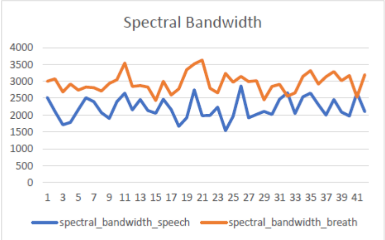

(d) *Spectral bandwitdth for breaths and speeches chunks*

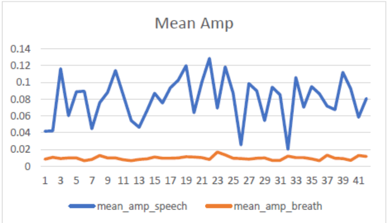

(e) *Mean amplitude for breaths and speeches chunks*

Figure S2: Comparison of the breathing and speech chunks. The breathing chunks are compared with the chunks of the speeches. The features (please refer to Supplementary Background 1.5 for the details) have been extracted (by python package Librosa) from these chunks separately. Only 5 of them have shown substantial differences in terms of the features' values between the breathing chunks and the speech chunks. They are as follows: [a] Spectral contrast graph for the breathing chunks and the speech chunks; [b] Roll off at 95% for for the breathing chunks and the speech chunks; [c] Root mean squared energy deviation for the breathing chunks and the speech chunks; [d] Spectral bandwidth for the breathing chunks and the speech chunks; [e] Mean amplitude for the breathing chunks and the speech chunks.

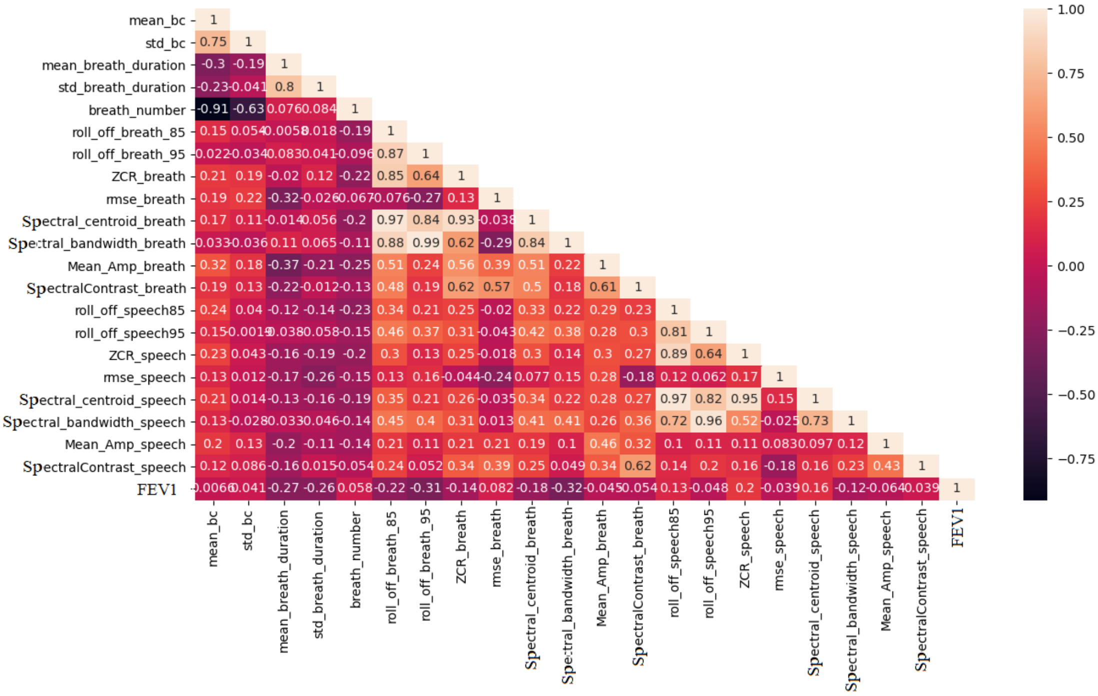

Figure S3: Correlations between features extracted from speech and breath parts. This figure represents the correlation of the features with each other. The Pearson correlation coefficient (PCC) has been calculated for each feature with others. Each box contains a correlation value between each other. The correlation values range from -1 to 1 where 1 means the features are highly correlated and -1 means they are also highly correlated but their correlation is negative.

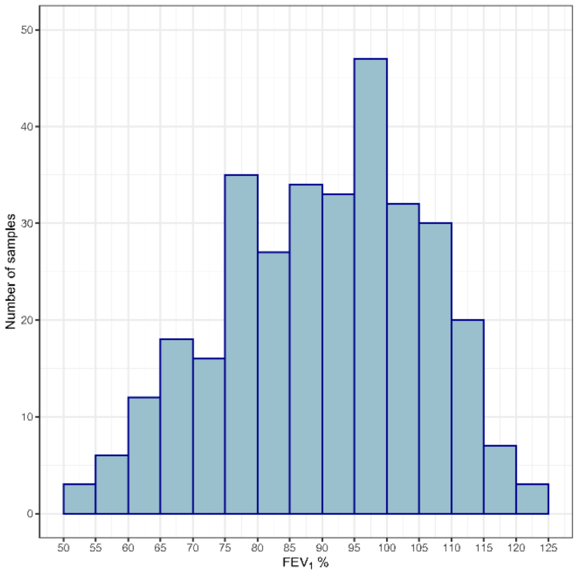

Figure S4: Histogram of FEV1% values of the samples. It represents the frequency distribution of FEV1% values of the samples. FEV1% values are represented in x-axis and frequency of the samples per FEV1% is shown in y-axis.

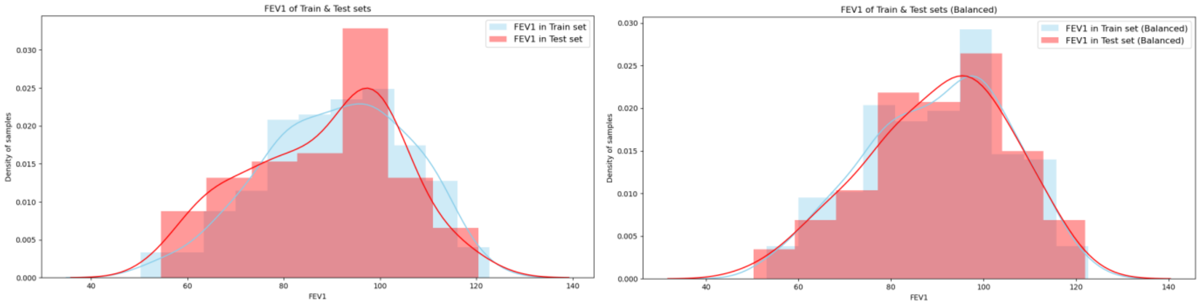

(A) (B)

Figure S5: The patterns of the training and the testing datasets. This figure represents the patterns of the training and the testing datasets. [1] the patterns of the training and the testing datasets without considering any special case. In this case samples are randomly separated for the training and testing sets at a ratio of 0.7:0.3 respectively. The distribution pattern of the samples on each FEV1% in the training and the testing sets show a small difference. [1] the patterns of the training and the testing datasets while the training and the testing samples are separately in a balancing way (please refer to the Section 4.1.4). In this case, samples are also randomly separated for the training and testing sets at the same ratio mentioned above. Additionally, the samples are separated at this ratio in each FEV1% range. The distribution pattern of the samples on each FEV1% range in the training and the testing sets present a similar pattern.

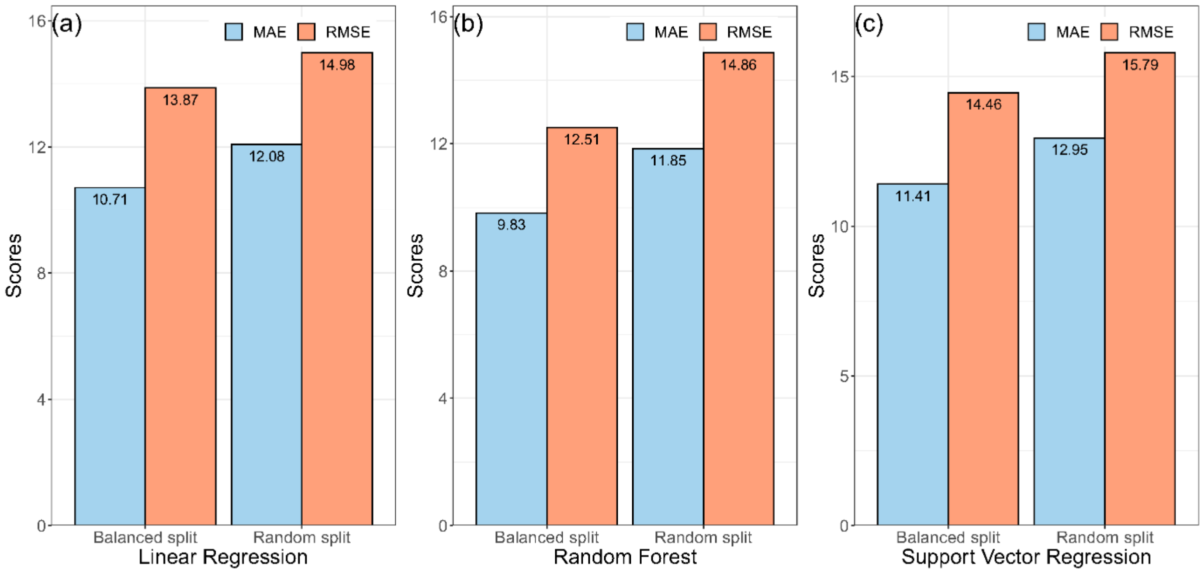

Figure S6: Effects of the balanced partitioning of the training and test sets. The performances of the $Model_1$[1], $Model_1$(RF) and $Model_1$(SVM), with and without balanced partitioning, are shown in (a), (b) and (c) respectively here. Balanced split represents the training and the test sets were created based sample distribution shown in Table 1 and the random split represent training and the test sets were created based on random partitioning. Here, MAE = Mean Absolute Error and RMSE = Root Mean Squared Error

4 Supplementary Tables

Table S1: Biological Attributes of 323 audio recordings

Biological attributes	Percentage/Mean	Standard Deviation
Sex (male)	86.38 %	
Height	176.10	8.20
Weight	81.27	21.48

Table S2: A threshold for separating the breath and speech chunks

Feature	Value
Spectral contrast	20.55
Roll-off at 95%	6000
Root mean squared energy	0.021
Spectral bandwidth	2300
Mean amplitude	0.018

Table S3: American Thoracic Society Grades for Severity of a Pulmonary Function Test Abnormality (FEV_1% = forced expiratory volume in one second percentage predicted.

Severity of lung function	FEV_1%
Normal	> 80
Mildly abnormal	70 to 79
Moderately abnormal	60 to 69
Moderate to severely abnormal	50 to 59
Severely abnormal	35 to 49
Very severely abnormal	< 35

Table S4: Percentage of the samples in terms of severity of abnormality of the lung function. Classification was done based on American Thoracic Society (ATS) grades. No samples were included from "severely abnormal" and "very severely abnormal" groups.

Severity of lung function	Percentage among samples
Normal	72.14 %
Mildly abnormal	15.78 %
Moderately abnormal	9.29 %
Moderate to severely abnormal	2.79 %

Table S5: Features extracted from the voice sound files

Macro Area	Feature
Breath Cycles Features	Mean breath cycle duration
	Breath cycle standard deviation
	Breaths number
Breaths Features	Mean breath duration
	Breath duration standard deviation
Breath and Speeches Features - Frequency domain	Roll off 85%
	Roll off 95%
	Zero Crossing Rate
	Root Mean Squared Energy
	Spectral Centroid
	Spectral Contrast
	Spectral Flatness
	Spectral bandwidth
	Mean Amplitude

Table S6: Performances of the $Model_1$ and $Model_{1P}$ based on Random Forest regression for predicting lung function. $Model_1$ used only features extracted from breath and speech part and $Model_{1P}$ included biological factors (sex, weight, and height) with the features in regression models and lung function was predicted in terms of FEV_1%. Here, MAE = Mean Absolute Error and RMSE = Root Mean Square Error.

Models	MAE	RMSE
$Model_{1(RF)}$	9.83	12.51
$Model_{1P(RF)}$	8.96	11.48

Table S7: Comparison of the performances of $Model_3$ in predicting the abnormality of lung function.

	Precision	Recall	F1-score	Accuracy	Sensitivity	Specificity	AUC
LR	0.77	0.78	0.76	0.78	0.37	0.94	0.78
RF	0.80	0.80	0.79	0.80	0.44	0.94	0.84
SVC	0.80	0.78	0.74	0.78	0.26	0.99	0.78

$Model_3$ used only features extracted from breath and speech parts in binary class classification models. Predicted binary classification was defined based on FEV1% classified either as normal (FEV1% < 80%) or abnormal (FEV1% ≥ 80%) based on the ATS definition of abnormal lung function. Here, RF = Random Forest, SVC = Support Vector Classifier and LR = Logistic Regression

Table S8: The performance in predicting normal vs. abnormal lung function resulted from the features from the voice features only ($Model_3$)

		Class label	Precision	Recall	F_1-score	Accuracy	Sensitivity	Specificity
RF	Abnormal	0	0.75	0.44	0.56			
	Normal	1	0.81	0.94	0.87			
	Macro average		0.78	0.69	0.72			
	Weighted average		0.80	0.80	0.79			
	Score		0.80	0.80	0.79	0.80	0.44	0.94
SVC	Abnormal	0	0.88	0.26	0.40			
	Normal	1	0.78	0.99	0.87			

	Macro average		0.83	0.62	0.63			
	Weighted average		0.80	0.78	0.74			
	Score		0.80	0.78	0.74	0.78	0.26	0.99
LR	Abnormal	0	0.71	0.37	0.49			
	Normal	1	0.80	0.94	0.86			
	Macro average		0.75	0.66	0.68			
	Weighted average		0.77	0.78	0.76			
	Score		0.77	0.78	0.76	0.78	0.37	0.94

Here, RF = Random Forest classifier, SVC = Support Vector Classifier and LR = Logistic Regression method

Table S9: Comparison of the performances of $Model_{3P}$ in predicting the abnormality of lung function.

	Precision	Recall	F1-score	Accuracy	Sensitivity	Specificity	AUC
LR	0.82	0.82	0.81	0.83	0.48	0.96	0.84
RF	0.84	0.85	0.84	0.85	0.63	0.93	0.88
SVC	0.81	0.80	0.78	0.80	0.37	0.97	0.79

$Model_{3P}$ used features extracted from breath and speech parts combined with biological factors (sex, weight, and height) in binary class classification models. Predicted binary classification was defined based on FEV1% classified either as normal (FEV1% < 80%) or abnormal (FEV1% ≥ 80%) based on the ATS definition of abnormal lung function. Here, RF = Random Forest, SVC = Support Vector Classifier and LR = Logistic Regression

Table S10: The performance in predicting normal vs. abnormal lung function resulted from the voice features with sex, weight and height ($Model_{3P}$)

		Class label	Precision	Recall	F_1-score	Accuracy	Sensitivity	Specificity
RF	Abnormal	0	0.77	0.63	0.69			
	Normal	1	0.87	0.93	0.90			
	Macro average		0.82	0.78	0.80			
	Weighted average		0.84	0.85	0.84			
	Score		0.84	0.85	0.84	0.85	0.63	0.93
SVC	Abnormal	0	0.83	0.37	0.51			
	Normal	1	0.80	0.97	0.88			
	Macro average		0.82	0.67	0.70			
	Weighted average		0.81	0.80	0.78			
	Score		0.81	0.80	0.78	0.80	0.37	0.97
LR	Abnormal	0	0.81	0.48	0.60			
	Normal	1	0.83	0.96	0.89			
	Macro average		0.82	0.72	0.75			
	Weighted		0.82	0.82	0.81			

average						
Score	0.82	0.82	0.81	0.83	0.48	0.96

Here, RF = Random Forest classifier, SVC = Support Vector Classifier and LR = Logistic Regression method.
